# Prognostic significance of fibroblast growth factor receptor 4 polymorphisms on biochemical recurrence after radical prostatectomy in a Chinese population

**DOI:** 10.1038/srep33604

**Published:** 2016-09-19

**Authors:** Luyao Chen, Zhengwei Lei, Xin Ma, Qingbo Huang, Xu Zhang, Yong Zhang, Peng Hao, Minggang Yang, Xuetao Zhao, Jun Chen, Gongxue Liu, Tao Zheng

**Affiliations:** 1 Department of Urology, Chinese PLA General Hospital, Beijing, China; 2 Department of Urology, Puai Hospital, Wuhan, China

## Abstract

Fibroblast growth factor receptor 4 (FGFR4) is a transmembrane receptor with ligand-induced tyrosine kinase activity and is involved in various biological and pathological processes. Several polymorphisms of FGFR4 are associated with the incidence and mortality of numerous cancers, including prostate cancer. In this study, we investigated whether the polymorphisms of FGFR4 influence the biochemical recurrence of prostate cancer in Chinese men after radical prostatectomy. Three common polymorphisms (rs1966265, rs2011077, and rs351855) of FGFR4 were genotyped from 346 patients with prostate cancer by using the Sequenom MassARRAY system. Kaplan–Meier curves and Cox proportional hazard models were used for survival analysis. Results showed biochemical recurrence (BCR) free survival was significantly affected by the genotypes of rs351855 but not influenced by rs1966265 and rs2011077. After adjusting for other variables in multivariable analysis, patients with rs351855 AA/AG genotypes showed significantly worse BCR-free survival than those with the GG genotype (HR = 1.873; 95% CI, 1.209–2.901; P = 0.005). Hence, FGFR4 rs351855 could be a novel independent prognostic factor of BCR after radical prostatectomy in the Chinese population. This functional polymorphism may also provide a basis for surveillance programs. Additional large-scale studies must be performed to validate the significance of this polymorphism in prostate cancer.

Prostate cancer is the most common malignancy and the second leading cause of cancer-related deaths in American males^1^. It is estimated that 233,000 new prostate cancer cases were diagnosed, and 29,480 deaths occurred in the United States in 2014^2^. Radical prostatectomy remains the first choice of treatment for patients diagnosed with clinically localized prostate cancer. Approximate 30% of these patients experience disease relapse as detected by the serum level of prostate-specific antigen (PSA)^3^. To date, the etiology and mechanism of prostate cancer remain unclear and poorly understood. Epidemiological data demonstrate that the incidence and mortality rate of prostate cancer vary in different countries and regions^2^,^4^,^5^, hence, the influence of racial or ethnic differences vary. Genome-wide association studies have identified genetic variants that are associated with risk or prognosis of prostate cancer, especially in European ancestry^6^,^7^,^8^. By contrast, few studies have investigated genetic variants that may be associated with prostate cancer in Asian ancestry. Apart from traditional clinicopathological indicators, including preoperative PSA level, Gleason score, and pathologic stage, new molecular biomarkers must be determined to improve the prediction of disease recurrence and progression; these markers could also provide a guide in making surveillance programs, especially after surgery.

Fibroblast growth factor receptor 4 (FGFR4) is a member of transmembrane receptors family with ligand-induced tyrosine kinase activity, which have variable affinities for different fibroblast growth factors (FGFs) and are involved in various biological processes including cell differentiation, development, hormonal signaling, and proliferative signaling^9^,^10^. Besides, FGF-FGFR signaling plays an important role in tumor growth and progression in several cancers^11^,^12^,^13^. Recent years many studies have focused on the clinical significance of polymorphisms of FGFR4, which are associated with the incidence and mortality of numerous cancers, such as lung cancer, gastrointestinal cancer, ovarian cancer, and melanoma^14^,^15^,^16^,^17^,^18^. Xu *et al*.^19^ performed a meta-analysis of prostate cancer by using six published papers and demonstrated that the polymorphism of FGFR4 (rs351855/Gly^388^Arg) contributed to prostate cancer incidence; the risk increased by 17% in the Arg^388^ allele compared with that in the Gly^388^ allele. FitzGerald *et al*.^20^ retrospectively evaluated data of 1458 American prostate cancer patients and found a weak association between rs351855 and prostate cancer-specific mortality. Ma *et al*.^21^ also showed that the polymorphisms of FGFR4 significantly influenced the development of prostate cancer in a Japanese population. Although genetic and ethnic factors may play an important role in the development of prostate cancer, the effect of the polymorphisms of FGFR4 on other racial populations with prostate cancer remains to be clarified.

In this study, we analyzed several common polymorphisms of FGFR4 in 346 Chinese men with clinically localized prostate cancer and investigated the prognostic role of these genetic variants in biochemical recurrence (BCR) after radical prostatectomy.

## Results

### Patient characteristics

The clinicopathological characteristics of patients are summarized in Table 1. The mean (±Standard Deviation) age of 346 patients with prostate cancer was 65.9 ± 7.0 years. During the mean 37.7 months and median 37.8 months of follow-up period, 124 (35.8%) patients experienced PSA recurrence and the remaining 222 (64.2%) patients did not. As shown in Table 1, Age and body mass index (BMI) were not significantly different between the two groups. By contrast, patients with BCR possessed higher PSA levels at diagnosis (P < 0.001), higher Gleason score (P = 0.001), higher proportions of advanced stage (P = 0.004) and positive surgical margin (P = 0.045) than those in patients without BCR.

### Survival analysis

**Figure 1:**
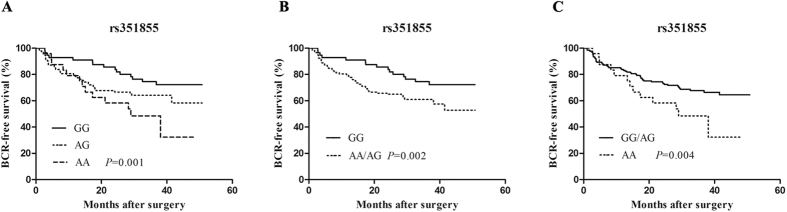
Kaplan-Meier survival analysis for BCR-free survival after radical prostatectomy according to FGFR4 rs351855 genotypes. (**A**) Three genotypes (**B**). Recessive model (**C**). Dominant model.

The genotypes of the three polymorphisms (rs1966265, rs2011077, and rs351855) of FGFR4 were identified, and their distribution in 346 patients is listed in Table 2. Kaplan–Meier survival analysis showed that apart from PSA (P < 0.001), Gleason score (P = 0.001), and pathologic stage (P = 0.005), the genotypes of rs351855 also significant influenced the BCR-free survival of patients with prostate cancer (P = 0.001, Fig. 1); by contrast, rs1966265 and rs2011077 did not influence the survival (P = 0.355 and P = 0.188, respectively, Supplemental Fig. 1). For rs351855, patients with the GG genotype showed significantly improved BCR-free survival, whereas patients with the AA genotype showed relatively worse BCR-free survival. Besides, BCR-free survival significantly differed in the recessive model (AA/AG vs GG, P = 0.002, Fig. 1) and dominant model (GG/AG vs AA, P = 0.004, Fig. 1). Multivariate analysis with hazard ratio (HR) and corresponding 95% confidence interval (CI) was further performed by adjusting for other variables including age, PSA, Gleason score, pathological stage, and margin status. The results showed that in recessive model patients with AA/AG genotypes had significantly worse BCR-free survival than those with the GG genotype (HR = 1.873; 95% CI, 1.209–2.901; P = 0.005; Table 2). In the dominant model, the differences were not significant (HR = 1.311; 95% CI, 0.828–2.076; P = 0.248). These data support that FGFR4 rs351855 may be an independent prognostic factor of BCR in prostate cancer after radical prostatectomy.

## Discussion

Single nucleotide polymorphisms (SNPs) are reported to be associated with cancer initiation or progression in various human malignancies, including prostate cancer. In this regard, genetic markers among cancer patients appears have been increasingly investigated. To date, several promising polymorphisms have not been used to screen for prostate cancer or incorporate into nomograms to predict patient survivals, however, may provide potential useful information to optimize the diagnosis, therapy and follow-up of patients with prostate cancer^22^,^23^,^24^.

In this study, three common polymorphisms of FGFR4 were successfully genotyped in 346 patients with clinically localized prostate cancer. The results showed a significant association between rs351855 (Gly^388^Arg) polymorphism and the BCR of prostate cancer in a Chinese population. Kaplan–Meier survival analysis revealed that patients with the rs351855 AA/AG genotype showed an unfavorable BCR-free survival than those with the GG genotype; the association remained significant even after adjusting for other clinicalpathological factors in the multivariable Cox model, which suggested the independent influence of rs351855 polymorphism on the BCR.

The underlying biological association between FGFR4 polymorphisms and prostate cancer has attracted extensive attention and several mechanisms have been proposed. Wang *et al*.^25^ established two human prostatic epithelial cell lines which predominantly express the FGFR4 Arg^388^ or Gly^388^ allele. Analysis of the colony morphology showed that cells expressing Gly^388^ grew tightly connected colonies, whereas cells expressing Arg^388^ grew in a scattered manner and had irregular morphology. Further migration and invasion assay suggested that Arg^388^ expression could significantly increase cell motility and invasiveness, which may be partially attributed to enhanced expression of the urokinase-type plasminogen activator receptor; this receptor is a key component of the urokinase plasminogen activator system and has been implicated in invasion and metastasis in various tumors, including prostate cancer^26^,^27^. Another study by Yu *et al*.^28^ demonstrated that the Arg^388^ variant showed increased receptor stability and sustained receptor activation after ligand binding compared with the Gly^388^ variant. Expression microarray analysis of prostatic epithelial cells lines expressing Arg^388^ or Gly^388^ was performed to investigate the influence of the sustained signaling on cellular signal transduction pathway. The expression of the Arg^388^ protein led to increased activity of the extracellular signal-related kinase (ERK) pathway, serum response factor, and transcription of multiple genes, which are correlated with aggressive clinical behavior in prostate cancer. Recently, Ulaganathan *et al*.^29^ proposed a novel mechanism and suggested that substitution of the conserved glycine 388 to a charged arginine can alter the transmembrane spanning segment and expose a membrane-proximal cytoplasmic signal transducer and activator of transcription 3 (STAT3) binding site, thereby enhancing STAT3 tyrosine phosphorylation by recruiting STAT3 proteins to the inner cell membrane. These findings may explain our results that patients with prostate cancer with the rs351855 A allele showed relatively poor BCR-free survival after radical prostatectomy.

To date, several groups have also evaluated the association between FGFR4 polymorphisms and prostate cancer prognosis, but their results are inconsistent. Wang *et al*.^25^ performed a retrospective analysis of 329 American patients with prostate cancer in 2004 and reported a statistically significant increase in the rate of PSA recurrence in patients with the Arg^388^ allele. Additionally, the presence of at least one FGFR4 Arg^388^ allele was significantly associated with the presence of lymph node metastasis at the time of surgery. These findings by Wang *et al*. suggest that the Arg^388^ allele is strongly associated with the occurrence of aggressive disease in American patients. By contrast, FitzGerald *et al*.^20^ evaluated 1458 patients with prostate cancer and 1352 controls; they suggested that none of the FGFR4 polymorphism genotypes are associated with prostate cancer risk or disease aggressiveness. However, when survival analyses were performed to evaluate the cancer-specific survival in the presence of FGFR4 polymorphism genotypes, the authors found that cases with the Arg^388^ allele experienced significantly worse cancer-specific survival relative to those with only the Gly^388^ allele (P = 0.04). Ma *et al*.^21^ also investigated the role of two common polymorphisms (rs2011077 and rs351855) in 492 Japanese patients with prostate cancer in 2008; patients with the AA genotype in rs351855 and the GG genotype in rs2011077 showed 1.8 and 5.6 fold increased risks of metastatic prostate cancer, respectively. In terms of cancer-specific survival and disease-free survival, no statistical significance was observed among the different genotypes of rs2011077 or rs351855, whereas rs351855 had an important prognostic role in the present cohort study. Hence, the present study had inconsistent results regarding the significance of FGFR4 polymorphisms, which may partially attributed to different baseline characteristics, treatments, and durations of follow-up employed. Noticeably, patients in previous studies have different races and varied frequency of alleles (e.g., Arg^388^ allele, 0.35 in whites and 0.11 in African-American in Wang *et al*., 0.31 in whites and 0.15 in African-American in FitzGerald *et al*., 0.47 in Japanese in Ma *et al*. and 0.41 in Chinese in our study). Therefore, environmental or genetic factors may influence the effects of polymorphisms.

To the best of our knowledge, this study is the first to evaluate the effect of FGFR4 polymorphisms on the BCR-free survival of Chinese patients with prostate cancer after radical prostatectomy. However, several limitations of our study must be acknowledged. First, our work is a retrospective study, with a relatively small population size of 346 patients compared with similar studies on other racial population. Second, the follow-up of our study is relatively short, which may have influenced the survival analysis. Besides, the endpoint employed in the study was only BCR-free survival without including disease-specific survival. Finally, apart from these three polymorphisms, some possible significant polymorphisms of FGFR4 might be missed. Thus, a large-scale study with comprehensive analysis of genetic variants of FGFR4 must be performed to validate the present results.

In summary, our work demonstrated that BCR-free survival significantly varied in patients with prostate cancer with different FGFR4 rs351855 genotypes. Hence, FGFR4 rs351855 may be a novel independent prognostic of BCR of Chinese patients with prostate cancer after radical prostatectomy. This functional polymorphism might provide an additional guide for design of surveillance programs. Additional large-scale studies are required to validate the significance of this polymorphism in prostate cancer.

## Methods

### Patient recruitment and data collection

In this study, 346 patients with clinically localized prostate cancer were recruited from the Chinese PLA General Hospital between January 2010 and December 2013. Patients in this cohort were diagnosed and pathologically confirmed to have prostate cancer after radical prostatectomy. The patients were followed up to evaluate the potential significance of genetic variants in the progression of prostate cancer. During the follow-up period, all patients were regularly instructed to have a series of PSA test every 3 months to monitor disease progression or recurrence. The PSA recurrence (BCR recurrence) was defined as two consecutive serial PSA measurements with >0.2 ng/mL at an interval of >3 months^30^ and the date of recurrence was set to the first follow-up with PSA level >0.2 ng/mL. To precisely analyze disease recurrence after radical prostatectomy, we excluded patients who received adjuvant hormone therapy or radiotherapy, as well as those lost to follow-up. According to the 7^th^ American joint committee on cancer (AJCC) tumor node metastasis (TNM) classification system of prostate cancer, the disease stage was determined by postoperative pathological examination, pelvic computed tomography or magnetic resonance imaging, and bone scan. The postoperative specimen was analyzed and pathologically graded based on the Gleason score system. The positive surgical margin was defined as tumor cells in the inked margin. This study was approved by the Institutional Review Board of the Puai Hospital and Chinese PLA General Hospital and was carried out in accordance with approved guidelines. Written informed consents were obtained from all included participants.

### SNPs genotyping

Whole blood samples of patients with prostate cancer were collected after admission and preserved in a freezer at −80 °C until analysis. Genomic deoxyribonucleic acid (DNA) was extracted from peripheral blood samples by DNA Blood Mini Kit (Qiagen, Valencia, CA, USA) according to the manufacturer’s protocol. SNPs of FGFR4 were selected based on previous relevant studies and the Hap-Map Chinese population data with a minor allele frequency (MAF) > 0.05. Finally, three SNPs (rs1966265, rs2011077, and rs351855) were included in the study and genotyped by the Sequenom MassARRAY iPLEX platform (Sequenom, Inc., San Diego, CA, USA) according to the manufacturer’s instructions. The primers used for each SNP are listed in Supplemental Table 1. For quality control, two blank controls in the 384-well plate were analyzed and 5% of the samples were randomly selected and repeated by DNA sequencing; the reproducibility was 100%.

### Statistical analysis

Clinicopathological characteristics in the BCR cohort and non-BCR cohort were compared by Student’s t-test (continuous variables) and the Chi-square test (categorical variables). The influence of genotypes on BCR-free survival interval was analyzed by the Kaplan–Meier method with log-rank test. The association between SNPs and PSA recurrence was assessed using different genetic models through multivariate Cox proportional hazards regression analysis with estimated HR and 95% CI, which were adjusted for other variables including age, PSA at diagnosis, pathologic stage, Gleason score and surgical margin. Statistical Package for the Social Sciences software version 19.0 (SPSS Inc., Chicago, IL.USA) was used for all analyses and a two-sided P < 0.05 was considered statistically significant.

## Additional Information

**How to cite this article**: Chen, L. *et al*. Prognostic significance of fibroblast growth factor receptor 4 polymorphisms on biochemical recurrence after radical prostatectomy in a Chinese population. *Sci. Rep.* **6**, 33604; doi: 10.1038/srep33604 (2016).

## Supplementary Material

Supplementary Information

## Figures and Tables

**Table 1:** Clinicopathological characteristics of prostate cancer patients who received radical prostatectomy.

Variables	Total	BCR	Non-BCR	*P* value
Patients, n	346	124	222	
Age, year, mean ± SD	65.9 ± 7.0	65.3 ± 7.2	66.2 ± 6.9	0.253
BMI, kg/m^2^**, n (%)**				0.277
<24	136(39.3)	43(34.7)	93(41.9)	
≥24	210(60.7)	81(65.3)	129(58.1)	
PSA, ng/ml, n (%)				**<0.001**
≤20	211(61.0)	56(45.2)	155(69.8)	
>20	135(39.0)	68(54.8)	67(30.2)	
Gleason score, n (%)				**0.001**
≤7	248(71.7)	76(61.3)	172(77.5)	
≥8	98(28.3)	48(38.7)	50(22.5)	
Pathological stage*, n (%)				**0.004**
Localized	250(72.3)	78(62.9)	172(77.5)	
Locally advanced	96(27.7)	46(37.1)	50(22.5)	
Margin status, n (%)				**0.045**
Positive	132(38.2)	56(45.2)	76(34.2)	
Negative	214(61.8)	68(54.8)	146(65.8)	
Follow up, months	mean 37.7, median 37.8			

Abbreviation: BCR, biochemical recurrence; BMI, body mass index; PSA, prostate-specific antigen; SD, standard deviation.

^*^TNM staging by 7^th^ AJCC, localized: T1/2N0M0, locally advanced: T3/4N1M0.

**Table 2:** Cox proportional hazards analysis of SNPs associated with PSA recurrence after radical prostatectomy.

SNPs	BCR, n (%)	Non-BCR, n (%)	Univariate analysis	*P* value	Multivariate analysis	*P* value
			HR(95% CI)		HR(95% CI)	
rs1966265						
GG	18(14.5)	43(19.4)	1.00			
AG	70(56.5)	112(50.5)	1.432(0.853–2.403)	0.175	1.226(0.724–2.076)	0.448
AA	36(29.0)	67(30.1)	1.213(0.689–2.137)	0.503	1.232(0.696–2.180)	0.474
AA/AG vs GG			1.349(0.818–2.224)	0.240	1.228(0.741–2.035)	0.425
AA vs GG/AG			0.922(0.626–1.359)	0.683	0.948(0.636–1.412)	0.792
rs2011077						
CC	19(15.3)	46(20.7)	1.00			
TC	71(57.3)	112(50.5)	1.591(0.949–2.667)	0.078	1.365(0.808–2.307)	0.245
TT	34(27.4)	64(28.8)	1.324(0.747–2.344)	0.336	1.297(0.730–2.303)	0.376
TT/TC vs CC			1.494(0.906–2.463)	0.115	1.341(0.810–2.220)	0.254
TT vs CC/TC			1.075(0.724–1.595)	0.719	0.975(0.651–1.459)	0.902
rs351855						
GG	30(24.2)	82(36.9)	1.00		1.00	
AG	68(54.8)	118(53.2)	**1.700(1.103**–**2.621)**	**0.016**	**1.842(1.169**–**2.902)**	**0.008**
AA	26(21.0)	22(9.9)	**2.637(1.553**–**4.477)**	**<0.001**	**1.968(1.122**–**3.452)**	**0.018**
AA/AG vs GG			**1.883(1.244**–**2.850)**	**0.003**	**1.873(1.209**–**2.901)**	**0.005**
AA vs GG/AG			**1.874(1.214**–**2.892)**	**0.005**	1.311(0.828–2.076)	0.248
