# Supplementary material for: Prognostic significance of fibroblast growth factor receptor 4 polymorphisms on biochemical recurrence after radical prostatectomy in a Chinese population
Source: Sci Rep. 2016 Sep 19;6:33604. doi: 10.1038/srep33604 (PMC5027536; doi:10.1038/srep33604)

Prognostic significance of fibroblast growth factor receptor 4 polymorphisms on biochemical recurrence after radical prostatectomy in a Chinese population

Luyao Chen[2], Zhengwei Lei[2], Xin Ma[2], Qingbo Huang[2], Xu Zhang[2], Yong Zhang[1], Peng Hao[1], Minggang Yang[1], Xuetao Zhao[1], Jun Chen[1], Gongxue Liu[1], Tao Zheng[1]*

[1] Department of Urology, Puai Hospital, Wuhan, China

[2] Department of Urology, Chinese PLA General Hospital, Beijing, China

[*] Corresponding author: Tao Zheng, M.D.

Department of Urology, Puai Hospital, Wuhan, 430033, China.

Tel: +86-027-68831681; Fax: +86-027-68831681

E-mail: tonyzt9007@sina.com

Supplemental Table 1. Primers for Sequenom allelic discrimination.

**Polymorphism**	**Sequence(5'-3')**
**rs1966265**	
1st PCR primer	TCCAGGGACAAGACTGGAG
2nd PCR primer	AGAGCTGTGAGAAGGAGATG
Extend primer	CAGGCACACTCAGCAGGA
**rs2011077**	
1st PCR primer	TCTGTGTCAGCTTTGACTTC
2nd PCR primer	CCATAAGGAAGGAGGTAGAG
Extend primer	AGCTTTGACTTCACATCTC
**rs351855**	
1st PCR primer	AAGCGGGAGAGCTTCTGCAC
2nd PCR primer	CTTGGCTGTGCTCCTGCTG
Extend primer	TCCCGCCCTCGATACAGCC

Supplemental Figure 1. Kaplan-Meier survival analysis for BCR-free survival after radical prostatectomy according to FGFR4 rs1966265 and rs2011077 genotypes

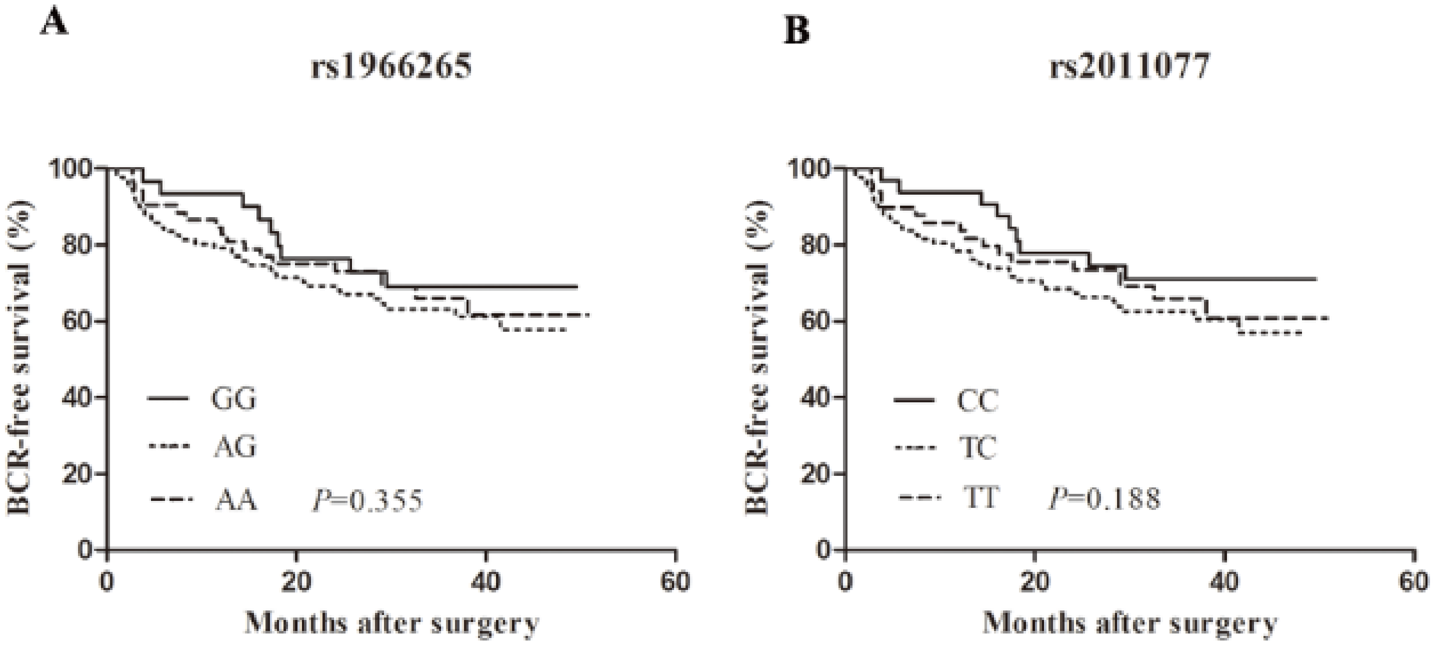

Supplement: Supplementary Information [file srep33604-s1.pdf]
